# Exploring the fragmentation efficiency of proteins analyzed by MALDI-TOF-TOF tandem mass spectrometry using computational and statistical analyses

**DOI:** 10.1371/journal.pone.0299287

**Published:** 2024-05-03

**Authors:** Jihyun Park, Clifton K. Fagerquist

**Affiliations:** 1 Western Regional Research Center, Agricultural Research Service, USDA, Albany, CA, United States of America; 2 U.S. Department of Energy, Research Participation Program Administered by the Oak Ridge Institute for Science and Education, Oak Ridge, TN, United States of America; Fisheries and Oceans Canada, CANADA

## Abstract

Matrix-assisted laser desorption/ionization time-of-flight-time-of-flight (MALDI-TOF-TOF) tandem mass spectrometry (MS/MS) is a rapid technique for identifying intact proteins from unfractionated mixtures by top-down proteomic analysis. MS/MS allows isolation of specific intact protein ions prior to fragmentation, allowing fragment ion attribution to a specific precursor ion. However, the fragmentation efficiency of mature, intact protein ions by MS/MS post-source decay (PSD) varies widely, and the biochemical and structural factors of the protein that contribute to it are poorly understood. With the advent of protein structure prediction algorithms such as Alphafold2, we have wider access to protein structures for which no crystal structure exists. In this work, we use a statistical approach to explore the properties of bacterial proteins that can affect their gas phase dissociation via PSD. We extract various protein properties from Alphafold2 predictions and analyze their effect on fragmentation efficiency. Our results show that the fragmentation efficiency from cleavage of the polypeptide backbone on the C-terminal side of glutamic acid (E) and asparagine (N) residues were nearly equal. In addition, we found that the rearrangement and cleavage on the C-terminal side of aspartic acid (D) residues that result from the aspartic acid effect (AAE) were higher than for E- and N-residues. From residue interaction network analysis, we identified several local centrality measures and discussed their implications regarding the AAE. We also confirmed the selective cleavage of the backbone at D-proline bonds in proteins and further extend it to N-proline bonds. Finally, we note an enhancement of the AAE mechanism when the residue on the C-terminal side of D-, E- and N-residues is glycine. To the best of our knowledge, this is the first report of this phenomenon. Our study demonstrates the value of using statistical analyses of protein sequences and their predicted structures to better understand the fragmentation of the intact protein ions in the gas phase.

## Introduction

Top-down proteomic (TDP) analysis involves the identification of the mature sequence and post-translational modifications (PTM) of undigested proteins using mass spectrometry (MS), tandem mass spectrometry (MS/MS) and a variety of gas phase dissociation techniques. These dissociation techniques include collision-induced dissociation (CID) [1], collision-activated dissociation (CAD) [2], high energy dissociation (HCD) [3], sustained-off-resonance irradiation (SORI)-CAD [4], surface-induced dissociation (SID) [5], in-source decay (ISD) [6], post-source decay (PSD) [7], blackbody infrared radiative dissociation (BIRD) [8], ultraviolet photodissociation (UV-PD) [9], electron capture dissociation (ECD) [10], electron transfer dissociation (ETD) [10], and many others. These dissociation techniques can be broadly grouped as either ergodic or non-ergodic. Ergodic techniques (CID, CAD, SORI-CAD, HCD, SID, PSD, BIRD) involve depositing energy into a protein ion in the gas phase such that it is redistributed amongst all the rotational/vibrational modes of the molecule over a timescale of microseconds (μs), milliseconds (ms), or seconds (s) after which the metastable protein ion dissociates, resulting in detectable fragment ions. Non-ergodic techniques (ECD, ETD, UV-PD, ISD) involve bond cleavage as a resultof proton/electron recombination or by absorption of UV photons. Unlike ergodic dissociation techniques, non-ergodic techniques have the advantage that PTMs attached at residue side-chains can be localized to specific residues, whereas ergodic techniques may result in dissociative loss of the attached PTM before its location has been determined definitively.

Electrospray ionization (ESI) is generally favored for TDP analysis as it results in multiply charged (protonated) higher charge state protein ions bringing the mass-to-charge (*m/z*) of protein ion within the *m/z* range of most mass analyzers as well as increasing coulomb repulsion during gas phase dissociation and facilitating electron/proton recombination reactions integral to ECD, ETD, and ISD [11]. The other soft ionization technique, matrix assisted laser desorption/ionization or MALDI [12], has found use for TDP analysis in taxonomic identification of bacterial microorganisms and mass spectrometry imaging (IMS) [13]. MALDI is frequently (although not exclusively) coupled to time-of-flight (TOF) mass analyzers for analyzing low charge protein ions generated by MALDI [14]. When MALDI is coupled with TOF and tandem TOF or TOF-TOF platforms, there are some limitations that restrict its use for TDP analysis. First, there are a relatively small number of dissociation techniques: ISD, high energy CID and PSD. Second, these platforms have limited resolution and mass accuracy compared to other mass analyzers, e.g. Orbitrap and FT-ICR. Third, ion isolation for MS/MS has limited resolution, as it relies on spatially separating Gaussian-shaped ion packets based on their arrival time at a mass gate. Fourth, switching rapidly from MS to MS/MS mode is currently not possible. In spite of these limitations, MALDI-TOF-TOF has some attractive features for TDP analysis: generation of low charge state fragment ions (often +1) that are often easy to assign, analysis without prior sample fractionation such as liquid chromatography (protein ions can be resolved and isolated by the first TOF stage of TOF-TOF platforms for MS/MS), ease of MALDI sample preparation, and speed of data acquisition and analysis.

Our laboratory and others [15–20] have demonstrated the utility of MALDI-TOF-TOF and MS/MS-PSD in identifying non-digested protein biomarkers from complex unfractionated bacterial samples. Complex mixtures of proteins can be analyzed directly, allowing for rapid analysis. However, the fragmentation efficiency can vary widely amongst these low charge state protein ions. PSD is an ergodic dissociation technique that results in polypeptide backbone cleavage on the C-terminal side of aspartic acid (D), glutamic acid (E) and asparagine (N) residues as well as on the N-terminal side of proline residues (P), resulting in b-type and y-type fragment ions (as well as dissociative losses of water and ammonia) [18]. The mechanism of backbone cleavage is commonly referred to as the aspartic acid effect [21–24].

Some early studies have explored the gas phase dissociation of peptides [25] and intact proteins [21,26] by PSD. It is generally understood that many factors, such as the amino acid composition, sequence and size contribute to its fragmentation pattern and efficiency. Previous statistical analysis of factors affecting fragmentation (via MALDI TOF MS/MS and ESI ion trap MS/MS) has generally focused on the cleavage residue; for instance, the N-terminal adjacent residue and C-terminal adjacent residue [27–29] and the types of ions observed [27,28]. However, these studies were done within the context of bottom-up proteomics—on peptides and focused on CID.

Studies on the effects of intact protein properties regarding fragmentation efficiency by PSD is lacking compared to studies on peptides, presumably due to their more complex structure. In this work, we use a statistical approach to explore the effects of various properties of intact proteins on fragmentation efficiency by PSD. We identify fragment signals from MS/MS-PSD spectra of proteins analyzed via MALDI-TOF-TOF, compare the data to predicted MS/MS-PSD fragments and assign them a score based on their abundance. We then predict their corresponding protein structures and extract various structural and biochemical properties. In our analysis, we examine fourteen of these properties (ten numerical and four categorical) in relation to the signal score for D-, E-, N-residue fragments resulting from PSD.

## Materials and methods

### Sample preparation

Bacterial sample preparation and mass spectrometry data acquisition has been described in detail previously [15]. Handling of bacterial samples was performed in a Class II biohazard cabinet (Baker Company). Briefly, a bacterial strain was cultured on Luria-Bertani agar (ThermoFisher) overnight at 37°C in a static incubator. One to two μL of cells were harvested with a sterile 1 μL loop and transferred to 300 μL of extraction solution in a 2 mL, O-ring-lined, screw-cap microcentrifuge polypropylene microvials (Biospec Products, Bartlesville, OK). The extraction solution was either HPLC grade water (Fisher Chemical) or 33% acetonitrile (Fisher Chemical), 67% water and 0.2% trifluoroacetic acid (Sigma-Aldrich, St. Louis, MO). Approximately 30 mg of 0.1 mm diameter zirconia/silica beads (Biospec Products) were added to the tube. The tube was tightly capped and agitated with a mini-bead-beater for 2 minutes (Biospec Products). The tube was then centrifuged for 3 minutes at 13,000 rpm (Eppendorf, Germany).

### Mass spectrometry

1.5 μL of sample supernatant was spotted onto 384-spot stainless steel MALDI target (Sciex, Redwood City, CA) and allowed to dry. The dried sample spot was then overlayed with 1.5 μL of a saturated solution of sinapinic acid (Life Technologies, ThermoFisher) dissolved in a solution of 33% acetonitrile, 67% water and 0.2% trifluoroacetic acid. Redissolved sample with matrix was then allowed to dry.

MS and MS/MS data was collected on a 4800 MALDI-TOF-TOF mass spectrometer (Sciex, Redwood City, CA) equipped with a pulsed solid-state YAG laser (λ = 355 nm, τ = 5 ns) with a 200 Hz repetition rate. MS data was collected in linear mode. After a brief delay (~1 μs) following the laser pulse, ions were accelerated from the source at 20.0 kV after which they strike the linear detector. The *m/z* range was 2000 to 20,000. MS data was collected, summed and signal averaged from 1000 laser shots. MS linear mode was externally calibrated with the +1 and +2 charge states of cytochrome-C, myoglobin and lysozyme (Sigma-Aldrich, St. Louis, MO).

MS/MS-PSD data was collected in reflectron mode wherein after a brief delay (~300 ns) following the laser pulse, ions were accelerated from the source at 8.0 kV. Upon reaching the timed-ion selector or TIS (a mass gate that selects the precursor ion based on its *m/z* and thus its arrival time), the selected precursor ion transits the TIS gate unimpeded where ions arriving outside the TIS window too soon or too late, are blocked. A typical TIS window is manually set to the precursor mass ± 100 Da. The TIS window was narrowed further, when necessary, to exclude fragment ions from neighboring protein ions if present. After the TIS, the mass-selected precursor ion was then decelerated to 1.0 kV after which it enters the collision cell. As no collision gas was introduced into the collision cell, any fragmentation is due to post-source decay (PSD), i.e. delayed fragmentation resulting from internal energy acquired by the ion during the ionization/desorption process in the source. After the collision cell, fragment ions and unfragmented precursor ion were re-accelerated to 15.0 kV. A metastable suppressor (another mass gate) was used to block any unfragmented precursor ion from advancing to the reflectron mirror to increase the detection sensitivity of fragment ions. Fragment ions were reflected nearly 180° by a 2-stage reflectron mirror: mirror #1: 10.515 kV and mirror #2: 18.330 kV) after which ions strike the reflectron detector. The MS/MS *m/z* range spans from 9.0 to above (+500 to 1000) the *m/z* of the precursor ion. MS/MS data was collected, summed and signal averaged from 10,000 laser shots. MS/MS reflectron mode was externally calibrated with the PSD fragment ions of singly charged alkylated thioredoxin.

Data was viewed using Data Explorer® software (Version 4.9, *Sciex*, Redwood City, CA). Raw MS/MS data was processed in the following sequence: Advanced baseline correction (Baseline correction parameters: Peak width: 32; Flexibility: 0.5; Degree: 0.0), Noise removal (Std dev to remove: 2.00) and Gaussian smoothing (Filter width: 31 points). The processed MS/MS data was then centroided and exported as an ASCII spectrum consisting of two columns of data: *m/z* and absolute intensity. Processed and centroided MS/MS data are provided at https://github.com/jpark837/PSD.

### Extraction of protein properties

The protein properties analyzed in this work are sequence and structurally based. We used Alphafold2 (version 2.2.0) to predict the structure of each of the bacterial proteins using the default databases [30]. We then selected bacterial proteins that were pre-identified for which MS/MS-PSD data was available. We wrote a pipeline in python to extract 14 properties for each instance of either a D-, E-, or N-residue from the proteins. We used PyMol (Schrödinger) to count the number of intramolecular backbone and sidechain hydrogen bonds, as well as to check for a salt bridge presence for each residue instance. For hydrogen bonds, we considered electrostatic pairings of the protonated lysine (K) and arginine (R) residues with deprotonated aspartic acid (D) and glutamic acid (E) residues. We chose a bond length range under 4.0 Å for salt bridges [31].

Secondary structure assignment and relative solvent accessible surface area calculations were done using the DSSP program [32]. The remaining numerical properties (degree, clustering coefficient, closeness, betweenness, eigenvector centrality, eccentricity, average nearest neighbor degree and strength) are centrality measurements from residue interaction network (RIN) analysis [33]. We used the Network Analysis or Protein Structure (NAPS) webserver for prediction and centrality analysis of the RIN for each protein [34]. For the NAPS webserver, we used the following options: C-alpha network type, weighted, threshold of 0–7 Å, and residue separation of 1. For comparison between networks, we adjusted eccentricity to be normalized to the protein diameter [34]. The protein diameter is the maximum eccentricity value of the network.

Alphafold2 predicted protein structures and the code used to extract the structural properties and accompanying data are available at https://github.com/jpark837/PSD.

### Computational and statistical analyses

All Alphafold2 predictions were run on a GPU node through the USDA-ARS Scientific Computing Initiative (SCINet) Ceres high-performance computing (HPC) cluster.

All statistical analyses and plot generation was done using Python and R.

For multivariate regression analysis, we assumed the response variable *Y* (signal score) to follow a negative binomial distribution with a mean of *E[Y] = μ* and let *x*_*p*_ be a set of explanatory variables (extracted properties). *μ* is then related to the explanatory variables as Eq 1. We scaled the explanatory variables from 0–1 for comparative interpretation before fitting the linear model to our data containing the signal score and property values for each fragment.


$$log(\mu )=\beta_{0}+\sum_{i=1}^{p}\beta_{0}x_{i}$$ (1)

To analyze the significance of the categorical properties (secondary structure, N-terminal adjacent residue, C-terminal adjacent residue and salt bridge presence), we performed the Kruskal-Wallis test to check if any groups within each property deviates significantly. We then performed the pairwise Mann-Whitney U test to identify the group within each categorical property that was significantly different.

For analysis of the categorical properties (N-terminal adjacent residue and C-terminal adjacent residue), we used all 36 bacterial proteins, as they only depend on the protein sequence. For the remaining categories, we removed 3 bacterial proteins that had a poor average predicted local distance difference test (pLDDT) score below 70 (S1 Table), as these properties depend on the predicted protein structure from Alphafold2.

## Results

### Calculation of signal scores

**Fig 1:**
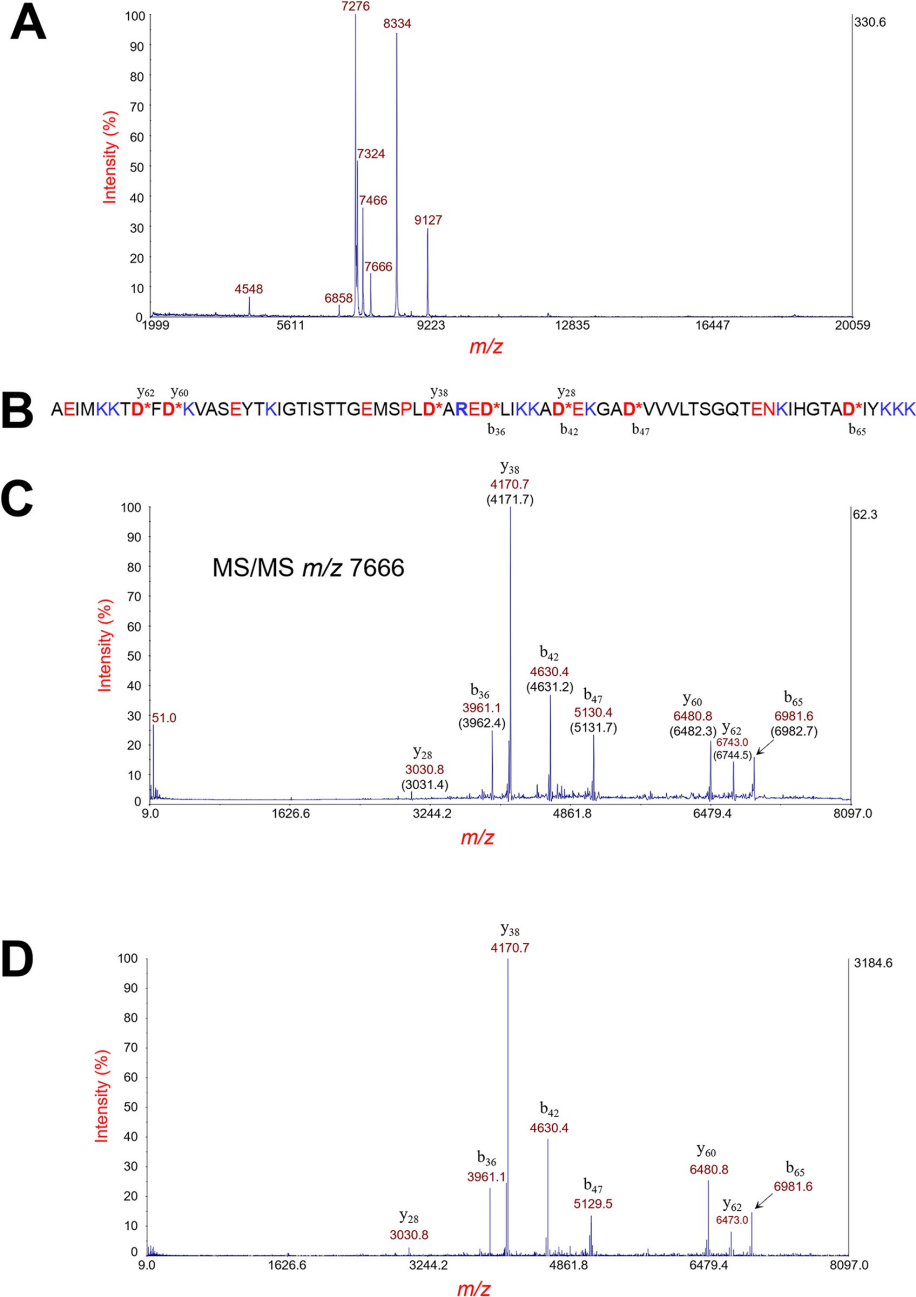
Example MS data of a of a strain of *Salmonella enterica* subsp. *enterica* serovar infantis. (A) Linear MS data of bacterial cell lysate. (B) The identified protein sequence (hypothetical/YahO) after removal of its 21-residue signal peptide. An asterisk denotes a site of backbone cleavage with its corresponding b-type and/or y-type fragment ions. (C) MS/MS data of the protein ion at *m/z* 7666. Fragment ions are identified by *m/z* (theoretical value in parentheses) and their b- or y-type fragment ion designation. (D) The pre-processed and centroided MS/MS data of the protein ion at m/z 7666. Pre-processed and centroided MS/MS data is shown in Fig 1D.

We selected 36 bacterial proteins for which MS/MS data was available for analysis (S1 Table and at https://github.com/jpark837/PSD). A typical example of MS and MS/MS data is shown in Fig 1 wherein a protein biomarker is identified from its intact mass by MS and its characteristic fragment ions obtained by MS/MS. Each protein in our study was previously identified by top-down proteomic analysis and confirmed by manual inspection comparing observed fragment ions to that of *in silico* fragment ions of the identified protein sequence. The *aspartic acid effect* is the dominant fragmentation mechanism of low charge state protein ions that fragment by PSD. Subsequently, the most prominent fragment ions are the result of backbone cleavage on the C-terminal side of D-, E- and N-residues and on the N-terminal side of P-residues, resulting in characteristic backbone b-type and y-type fragment ions. Isobaric protein ions, i.e. protein ions that have the same nominal *m/z* and are thus not isolatable from each other by our TIS mass gate, would result in a mixture of fragment ions from both protein ions. Such a circumstance was not observed in the 36 proteins analyzed in this study. All the fragment ions of each MS/MS experiment corresponded to a single protein sequence.

**Fig 2:**
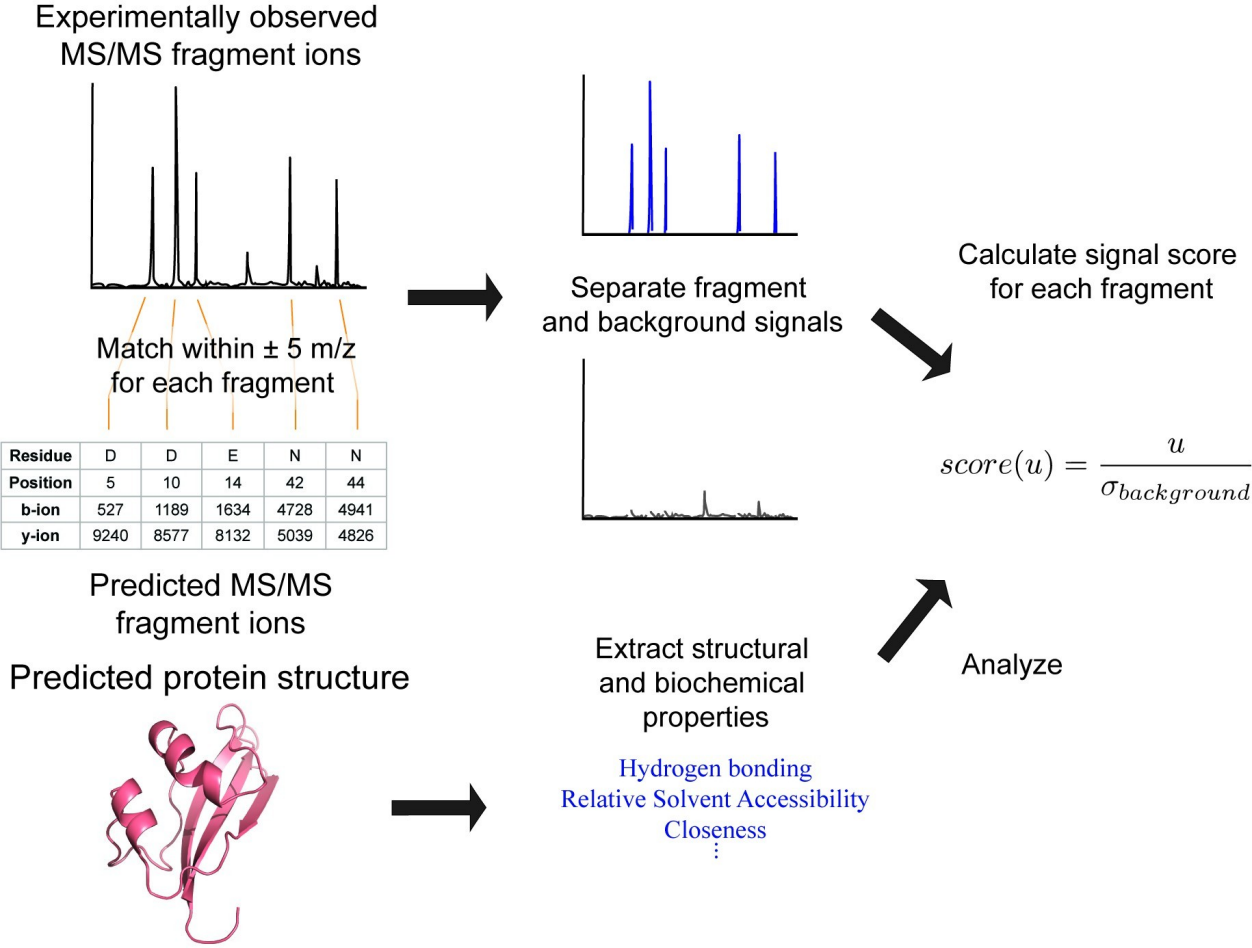
Workflow for analysis of the selected bacterial proteins.

The raw MS/MS data for each protein was processed, centroided and exported as an ASCII spectrum and analyzed (Fig 2). GPMAW (version 13.03) was used to predict the average *m/z* of b- and y-type fragment ions resulting from *in silico* backbone cleavage on the C-terminal side of D-, E and N-residues for each protein sequence [35]. *In silico* fragment ions generated by GPMAW are provided at https://github.com/jpark837/PSD. Our script then matched each predicted fragment ion to the highest signal intensity of the MS/MS data within ± 5 *m/z*. The script also accounted for loss of ammonia (-17 *m/z*) and water (-18 *m/z*) for each fragment ion to separate noise from background as much as possible. Once fragment signals were assigned and separated, our script compared the b- and y-type fragment ion intensity for each backbone cleavage position, then considered the larger of the two as the fragment signal (*u*).

For each fragment signal, we used Eq 2 to calculate a signal score. The signal score, which we defined as the ratio of the intensity of the fragment signal (*u*) and the standard deviation (*σ*) of the background (Eq 2), was our metric for fragmentation efficiency. A higher signal score indicates a higher likelihood of polypeptide backbone cleavage at that residue position, as the resulting fragment ion is more abundant. The standard deviation of the background was to normalize varying noise between MS/MS data.


$$score(u)=\frac{u}{\sigma_{background}}$$ (2)

### Backbone cleavage at E and N-residues have similar efficiencies

**Fig 3:**
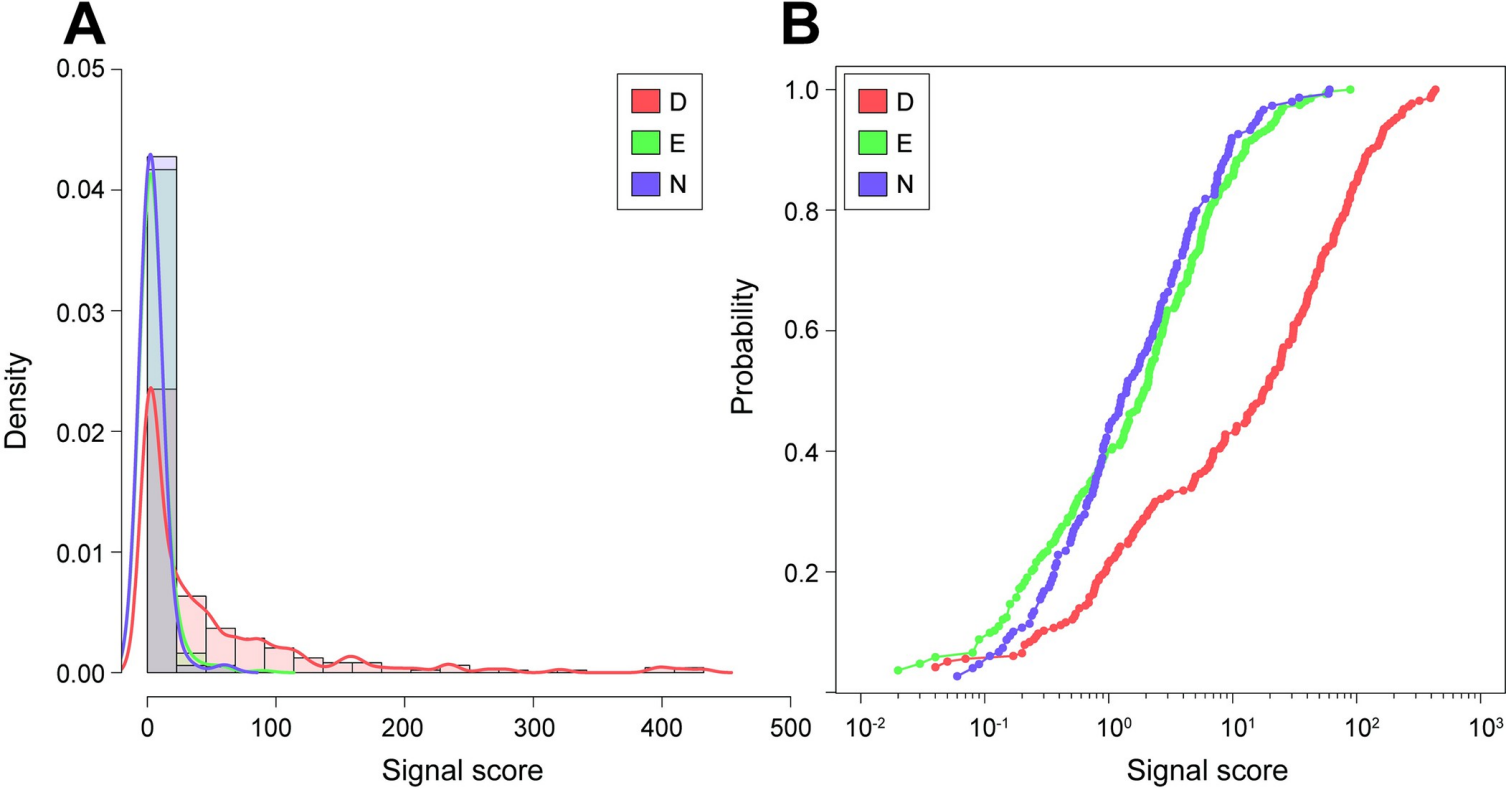
Distribution of D-, E-, and N-fragment signal scores. (A) Histogram of D-, E-, and N- fragment scores. (B) Empirical cumulative distribution functions of D-, E-, and N-fragment scores.

Initially, we noticed the distribution of our response variable, the signal score of each fragment, to overlap each other for E- and N-residues (Fig 3A). Plots of the empirical cumulative distribution function (eCDF) of signal scores for D-, E- and N-residues confirmed this observation, as we also saw the eCDFs of E- and N-residues to overlap (Pearson’s correlation coefficient = 0.99) (Fig 3B). This overlap indicates that E- and N-fragments have a similar spread of signal scores. In contrast, the eCDF of D-residues was distinct from E- and N-residues in that they were shifted towards the right, as a larger proportion of D-fragments have higher signal scores. For instance, ~56% of D-residue fragments have a signal score higher than 10 while for E- and N-residues, only ~15% of fragments do (Fig 3B). Together, our results suggest that polypeptide backbone cleavage on the C-terminal side of E- and N-residues have similar efficiencies and are lower than D-residues.

### Regression analyses reveal several centrality measures to be significant factors

We also noticed that the signal score for all residues were non-normal and heavily positively skewed (Fig 3A). This shape is characteristic of count-based data, for which there exist discrete probability distributions that provide convenient models for analysis [36,37]. We rationalized that by viewing the polypeptide backbone cleavage as an event with a probability of success, we could apply these types of models for our case [36]. The clustering of signal scores of D-, E- and N-fragments near 0, alongside extreme outliers at high signal scores, indicates overdispersion (Fig 3A). For protein properties which were numerical (Table 1), we consequently used negative binomial regression to assess the effect of each property on the signal score. The negative binomial distribution allows its variance to differ from its mean, allowing greater flexibility in handling dispersion [38].

**Table 1:** Multivariate regression analysis results.

	Aspartic acid (D)		Glutamic acid (E)		Asparagine (N)	
Explanatory variable	Coefficient Estimate ± standard error	P-value	Coefficient Estimate ± standard error	P-value	Coefficient Estimate ± standard error	P-value
**Degree**	0.46 ± 1.27	0.72	-1.62 ± 0.91	0.08	-2.59 ± 1.41	0.07
**Backbone hydrogen bond counts**	0.76 ± 0.70	0.28	0.58 ± 0.36	0.11	2.74·10^−4^ ± 0.77	1.00
**Sidechain hydrogen bond counts**	0.75 ± 0.60	0.21	0.80 ± 0.39	0.04	0.39 ± 0.54	0.47
**Relative solvent accessibility**	2.38 ± 0.89	7.42·10^−3^	1.30 ± 0.81	0.11	-1.50 ± 0.79	0.06
**Clustering coefficient**	-1.03 ± 0.73	0.16	-0.70 ± 0.67	0.30	1.17 ± 0.66	0.08
**Closeness**	4.48 ± 0.57	4.13·10^−15^	2.66 ± 0.46	8.31·10^−9^	1.17 ± 0.69	0.09
**Betweeness**	-1.69 ± 1.12	0.13	-1.38 ± 0.83	0.10	-0.09 ± 0.96	0.93
**Eigenvector centrality**	0.44 ± 0.69	0.52	-1.13 ± 0.57	0.05	1.52 ± 0.69	0.03
**Average nearest neighbor degree**	0.34 ± 1.02	0.74	1.55 ± 0.73	0.03	-1.17 ± 1.11	0.29
**Eccentricity**	2.38 ± 0.47	4.47·10^−7^	1.54 ± 0.39	6.92·10^−5^	0.64 ± 0.55	0.24
**Log-likelihood ratio test** **(model vs null)**	1.98·10^−12^		8.52·10^−12^		2.02·10^−5^	

Significant explanatory variables p<0.01 and p<0.05 are respectively highlighted in lavender and yellow.

A cross correlation matrix of the explanatory variables showed degree and strength to be strongly correlated with each other, as the pairwise Pearson’s r correlation coefficient between them was 1 (S1 Fig). We subsequently removed strength as an explanatory variable from our regression analysis to reduce redundancy. Our regression results for D-,E-, and N-residues are summarized in Table 1. We found various centrality measurements from residue interaction network (RIN) analysis to be significant. In RIN analysis, proteins are drawn as a network, where residues are considered as nodes while contacts between them are considered as edges [34].

**Fig 4:**
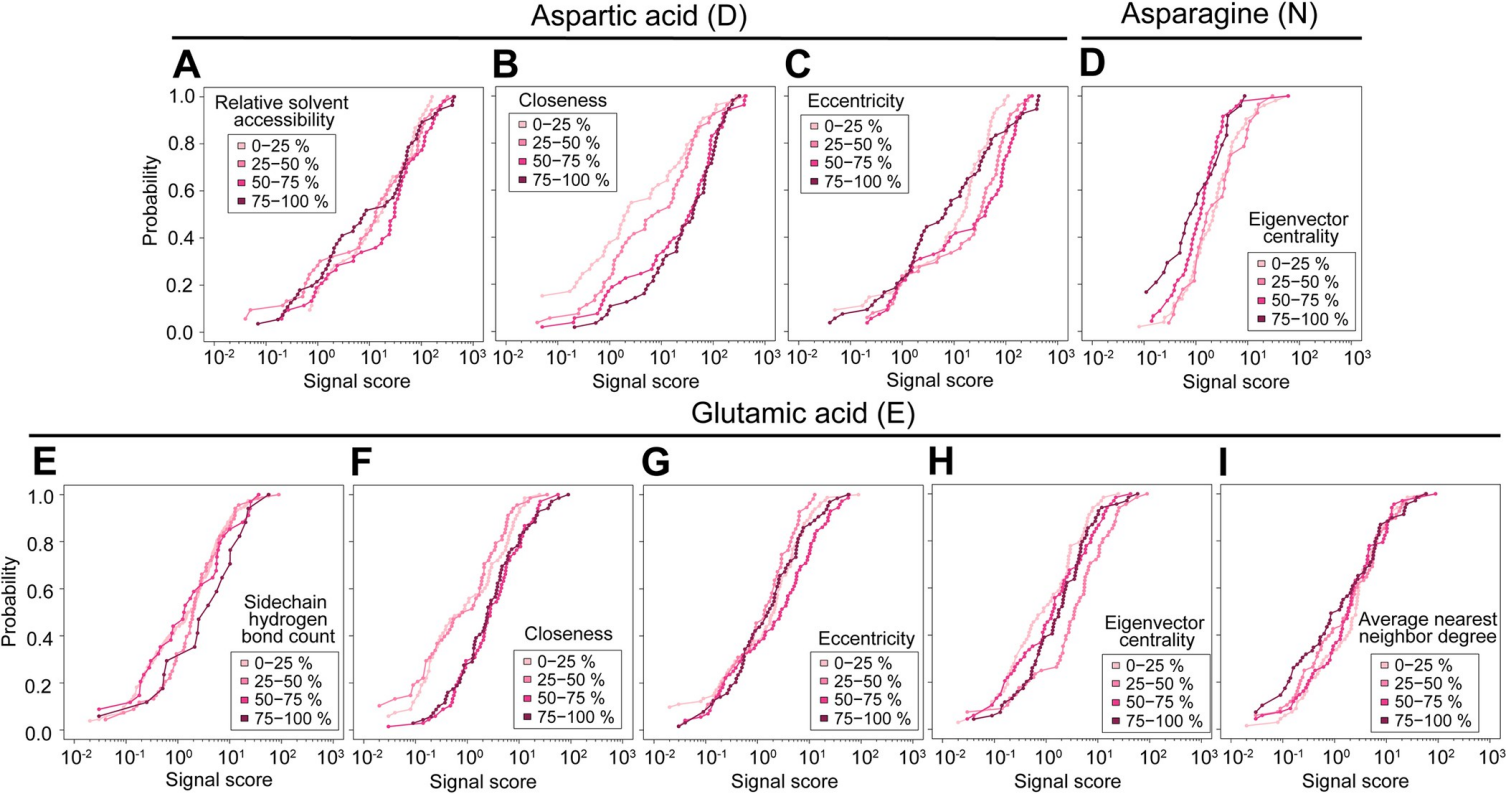
Distribution of D-, E-, and N- fragment signal scores with respect to significant explanatory variables. (A-C) Empirical cumulative distribution functions (eCDF) of D-fragment signal scores. (D) eCDF of N-fragment signal scores. (E-I) eCDF of E-fragment signal scores. Shades of pink represent percentile of significant explanatory variable values.

For D- residues, relative solvent accessibility, closeness, and eccentricity were significant (p<0.01) explanatory variables. Relative solvent accessibility describes how exposed or buried a residue is in a protein and is an important factor for determining its stability [39,40]. The positive value suggests that for D-, the less buried the residue is, the higher the signal score probability is up to a certain extent. D-fragments with relative solvent accessibility values that were in the 50–75% quartile had the highest distribution of signal scores. (Fig 4A).

Closeness is defined as the inverse of the shortest path distance (*dist(u*,*v)*) of a node (*n*) to all other nodes (*v*) (Eq 3). Closeness is an indicator of how close a node (residue) is to all other nodes in the network [34]. A positive coefficient estimate for closeness indicates that residues near other residues path wise are associated with a higher signal score probability, which we also clearly observed in its distribution (Fig 4B).


$$C_{Cl}(u)=\frac{n-1}{\sum_{uєV}dist(u,v)}$$ (3)

Eccentricity is defined as the shortest path distance of the residue to the farthest residue divided by the diameter of the protein (Eq 4). A higher value indicates the residue is closer to the periphery while a lower value indicates the residue is closer to the center [41]. The significant, positive coefficient estimate (p<0.01) for eccentricity indicates that D-residues that are closer to the periphery of the protein, but not at its absolute extremity leads to a higher signal score probability. For eccentricity, D-fragments with values that were the lowest 0–25% and the highest 75–100% quartiles had lower distribution of signal scores compared to those within the 25–50% and 50–75% quartiles (Fig 4C).


$$C_{e}(u)=\frac{max(dist(u,v))}{{diameter}_{protein}}$$ (4)

For E-sidechain hydrogen bond count, closeness, eccentricity, eigenvector centrality, and average nearest neighbor degree were significant (p<0.05) explanatory variables. Sidechain hydrogen bond count is the number of potential hydrogen bonds the sidechain of a residue is involved in within a bond length range between 2.5 and 3.2 Å. E-residues with the highest number of sidechain hydrogen bond counts (75–100% quartile) had the highest distribution of fragment signal scores (Fig 4E). Like D-, E-residues also had a positive coefficient estimate and distribution pattern for closeness (Fig 4F). Similarly for eccentricity, E-fragments that were the highest 75–100% quartiles had the highest distribution of signal scores (Fig 4G).

Eigenvector centrality is the eigenvector (*x_i_*) that corresponds to the largest eigenvalue (*λ*) of the adjacency matrix (*A_ij_*) [34,42] (Eq 5). This centrality metric indicates how connected a node is to other well-connected nodes in the network [34]. The negative coefficient estimate is reflected in its distribution, where E-fragments with eccentricity values in the 25–50% quartiles had the highest distribution of signal scores (Fig 4H).


$$x_{i}=\frac{1}{\lambda }\sum_{j=1}^{N}A_{ij}x_{j}$$ (5)

Average nearest neighbor degree is the average of the degree (*C_d_*(*u*)) of a node’s direct neighbors (*N*(*u*)) (Eq 6) [34]. This centrality metric quantifies the dependency between degrees of a node and its neighbors [43]. Although the variable was significant (p<0.05) and its coefficient estimate was positive (Table 1), we did not see a clear pattern upon visual inspection of the distribution of E-fragment signal scores with respect to average nearest neighbor degree (Fig 4I).


$$C_{an}(u)=\sum_{vєN(u)}\frac{C_{d}(u)}{N(u)}$$ (6)

For N-residues, only eigenvector centrality was a significant explanatory variable (p<0.05). The coefficient estimate for this variable was positive (Table 1). However, we saw that N-fragments with degree values in the lower 0–25% and 25–50% quartiles had higher distributions of signal scores (Fig 4D), indicating a negative relationship. The lack of significant explanatory variables closeness and eccentricity of N- compared to D- and E- is also interesting. The presence of an amide rather than a carboxylic acid on the side chain may present different behaviors regarding the aspartic acid effect.

### Presence of an adjacent C-terminal proline enhances fragmentation

**Fig 5:**
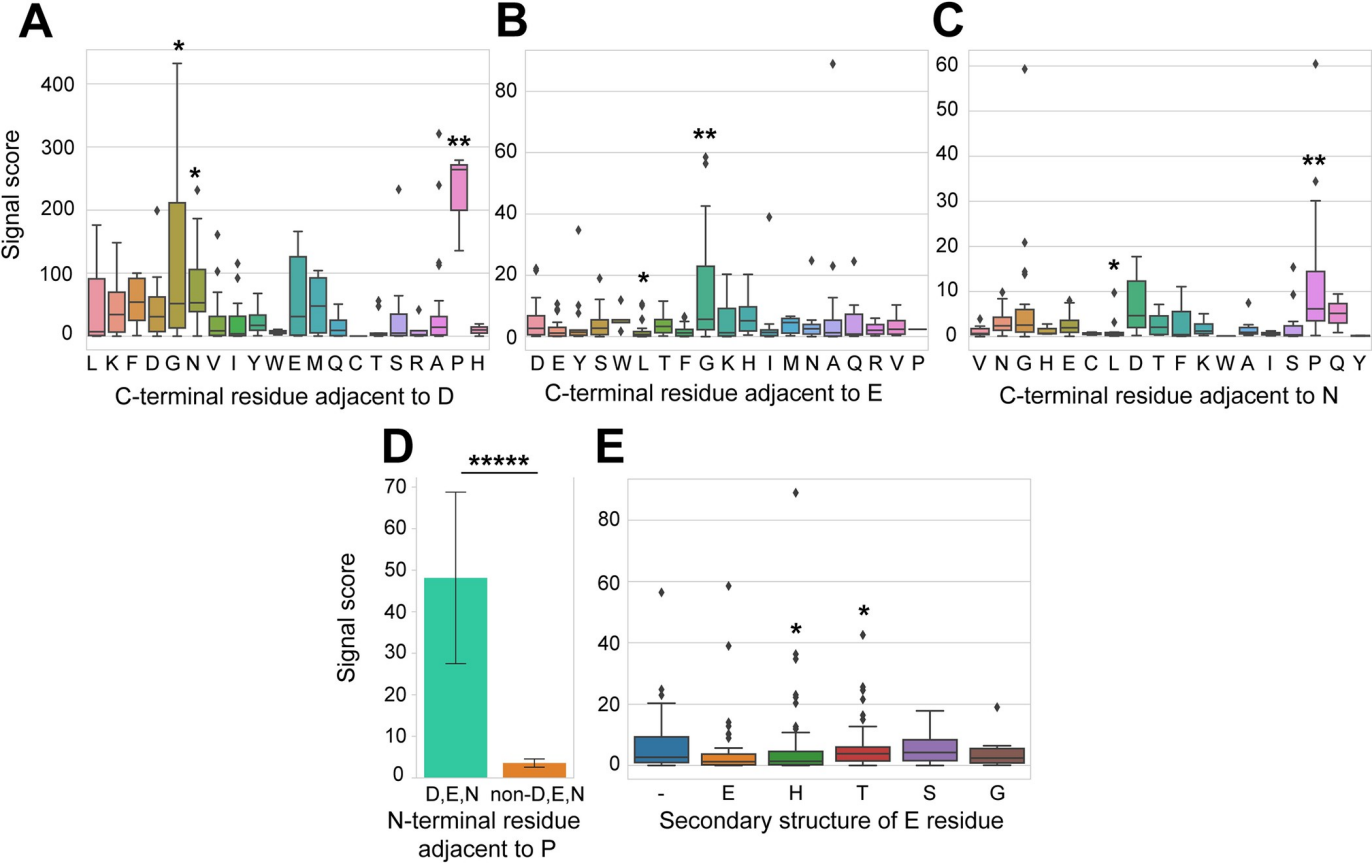
Analysis of categorical explanatory variables. (A) Box plot of fragment signal scores grouped by C-terminal residues adjacent to D. (B) Box plot of fragment signal scores grouped by C-terminal residues adjacent to E. (C) Box plot of fragment signal scores grouped by C-terminal residues adjacent to N. (D) Bar graph comparing the proline fragment signal scores whose adjacent N-terminal adjacent residue was D,E,N or non-D,E,N. (E) Box plot of fragment signal scores grouped by secondary structure of E-residues. Significant explanatory variables p<0.05, p<0.01, p<0.00001 is respectively marked by *,**, and ***** based on the Mann-Whitney U test. Bar graph is displayed as mean ± standard error.

We also analyzed four categorical properties, where we found the C-terminal adjacent residue to be a significant explanatory variable for all three residues (Table 2). The D-G, D-N, D-P, E-L, E-G, N-L, and N-P sequence motifs were found to be significant (p<0.05). Except for the E-L and N-L sequence motifs, the rest led to a higher signal score **(**Fig 5A–5C). We noticed that when P was present on the C-terminal side of D- and N-residues, the signal score of the fragments were dramatically higher. Indeed, for P-residue fragment ions, the presence of either a D-, E-, or N-residue on the N-terminal side significantly (p<0.00001) led to a higher signal score (48.1 ± 20.1). In contrast, P-residue fragment ions that did not have an adjacent N-terminal D-, E-, or N-residues had a lower signal score of 3.6 ± 1.0 (Fig 5D). E-residue alone did not show the E-P sequence motif to be significant, presumably because there was only one instance of the sequence motif in our dataset.

**Table 2:** Kruskal-Wallis test of categorical explanatory variables.

Explanatory variable	P-value		
	Aspartic acid (D)	Glutamic acid (E)	Asparagine (N)
**Secondary structure**	0.76	0.02	0.07
**N-terminal adjacent residue**	0.09	0.39	0.25
**C-terminal adjacent residue**	0.01	0.05	1.85·10^−3^
**Salt bridge presence**	0.37	0.15	NA^α^

Significant explanatory variables p<0.05 and p<0.01 based on the Kruskal-Wallis test are highlighted in yellow and lavender, respectively.

^α^Salt bridge was not predicted to be present on any N-residue.

For glutamic acid, the secondary structure assignment of the residue was also significant (Table 2). T, which stands for turn and designates single helix hydrogen bonds in DSSP, lead to a significantly higher signal score (Fig 5E). In contrast, H, which stands for a 4 residue-turn alpha helix, was significantly lower (Fig 5E) [32,44].

## Discussion

The aspartic acid effect is initiated by the transfer of a proton from a carboxylic acid or amide side-chain group to the backbone amine (S2 Fig) [24]. Comparing the gas-phase acidities (ΔG_*gas*_) of the side-chain carboxylic or amide hydrogen from aspartic acid (325.9 kcal/mol), glutamic acid (324.3 kcal/mol) and asparagine (332.7 kcal/mol) [45], we were surprised to find that our distribution of D-, E-, and N-fragment scores did not match this order. Instead, we observed that the efficiency of the C-terminal cleavage at E- and N- residues via PSD were nearly the same and lower than the cleavage efficiency at D-residues (Fig 3B). Alternatively, a combination of the side chain acidity, the basicity of the neighboring amine/imine (presence or absence of a proline), and the length of the side chain could explain the differing abundances between D-,E-, and N- fragments. For instance, although glutamic acid has a more acidic carboxylic proton than asparagine (which has an amide), it has nearly the signal score distribution (Fig 3B). Glutamic acid’s side chain is 1 carbon longer, which could deter the rearrangement required for the carboxylic proton to be in closer proximity to the neighboring backbone amine/imine. Aspartic acid has the highest signal score distribution, as it benefits from having a higher side chain acidity (carboxylic proton) and a shorter side chain length. Now consider glutamine, which suffers from both the side chain being less acidic (amide) and having a longer side chain. Although fragmentation at glutamine can occur [16], they are rare and seldom seen [46].

From our regression analyses, our results highly suggest that the local structural properties of proteins can affect fragmentation efficiency. For D- and E-residues, closeness was a highly significant (p<0.01) explanatory variable with a positive coefficient, indicating that residues that are near other nodes distance-wise are associated with a higher signal score probability. This could possibly be explained by a higher efficiency of distribution of internal energy. A residue with shorter interaction paths could allow for more energy transfer with less travel time [47]. Investigations into the energetics of metastable protein ions post-source would undoubtedly be insightful. In addition, for D- and E-residues, eccentricity was also highly significant (p<0.01), indicating that residues closer the periphery of the protein (although not at the extremity of the periphery) have a higher chance of fragmenting in comparison to those near the center.

We also showed that the presence of P-residues on the C-terminal side of either D- or N-residues dramatically enhances backbone cleavage. The D-P sequence motif is documented in peptides as well as proteins [21], and our results show that this motif can be extended to N-residues [48]. For now, we can only speculate the reason for this enhancement. P-residue is unique in that it is an imino acid–its backbone nitrogen is encircled with its side chain. P-residue can be a proton acceptor and an imine could have higher basicity than an amine in the gas-phase, as it has theoretically been shown in DMSO (S2B Fig) [46]. The cyclical nature of P-residues also renders them structurally very rigid, and it has been proposed as a disruptor of secondary structures [49,50]. The presence of proline may provide a local environment beneficial for cleavage. It is also possible that the cyclic structure of proline may obstruct efficient transfer of internal energy along the backbone. For instance, an internal energy bottleneck may result in an enhancement of the side-chain rearrangement of D- and N-residues when they are located on the N-terminal side of a P-residue.

## Conclusions

Three decades have passed since Yu *et al*.*’s* first description of the aspartic acid effect mechanism in protein ions generated by MALDI [21]. MALDI, coupled with TOF and TOF-TOF platforms has adaptable applications in high-throughput proteomics, especially in that of rapid protein identification. Despite the demonstrated use of MALDI TOF-TOF in proteomics, the structural and biochemical properties of proteins that affect their dissociation is relatively under-examined and poorly understood. We explore this topic in the context of bacterial proteins using new technologies. Our work highlights the local structural and sequence-based properties that affect their fragmentation via PSD, the main dissociation technique for MS/MS of intact protein ions from unfractionated protein mixtures on MALDI-TOF-TOF instruments for which no collision gas is used. The fragmentation bias we observe in this work potentially adds another dimension of the structural and sequence-based information from the proteins researchers identify and analyze. Moreover, our results may be applicable to other MS platforms that can generate low charge state protein ions fragmented by an ergodic dissociation technique as these ionization/dissociation conditions favor the aspartic acid effect fragmentation mechanism. Although our results were obtained within the context of an ergodic dissociation technique, such an analysis may also be useful in the study of gas phase protein ion structures and their fragmentation using non-ergodic dissociation techniques [9,10].

With recent advances in algorithms to reliably predict protein structures, it is important to utilize and further develop rapid mass spectrometry techniques that can confirm theoretical structures. Top-down proteomic analysis, native state mass spectrometry, H/D exchange mass spectrometry and ion mobility mass spectrometry are likely to be the most relevant gas phase techniques for making comparisons to *in silico* predicted structures, as the mature intact protein have been shown to be retained into the gas phase under certain conditions. Our current work seeks to extract various protein properties from Alphafold2 predictions and compare them to patterns of fragmentation observed for low charge state protein ions. This approach may be of value to other researchers pursuing mass spectrometry-based intact protein analysis whose goal, beyond identification, is structural elucidation.

## Supporting information

S1 Fig Cross-correlation matrix between the extracted numerical explanatory variables of D-, E-, and N-residues. Values represent Pearson’s correlation.
(TIF)

S2 Fig Comparison of the aspartic acid effect between D-residues with a C-terminal proline (P) residue (B) and those without (A). (A-B) The proposed aspartic acid effect mechanism [5]. (C) Theoretical pK_a_ values of amine and imine in DMSO [46].
(TIF)

S1 Table Bacterial proteins analyzed by MALDI-TOF-TOF-MS/MS. (DOCX)
