# Supplementary figures and images for: Exploring the fragmentation efficiency of proteins analyzed by MALDI-TOF-TOF tandem mass spectrometry using computational and statistical analyses
Source: PLoS One. 2024 May 3;19(5):e0299287. doi: 10.1371/journal.pone.0299287 (PMC11068200; doi:10.1371/journal.pone.0299287)

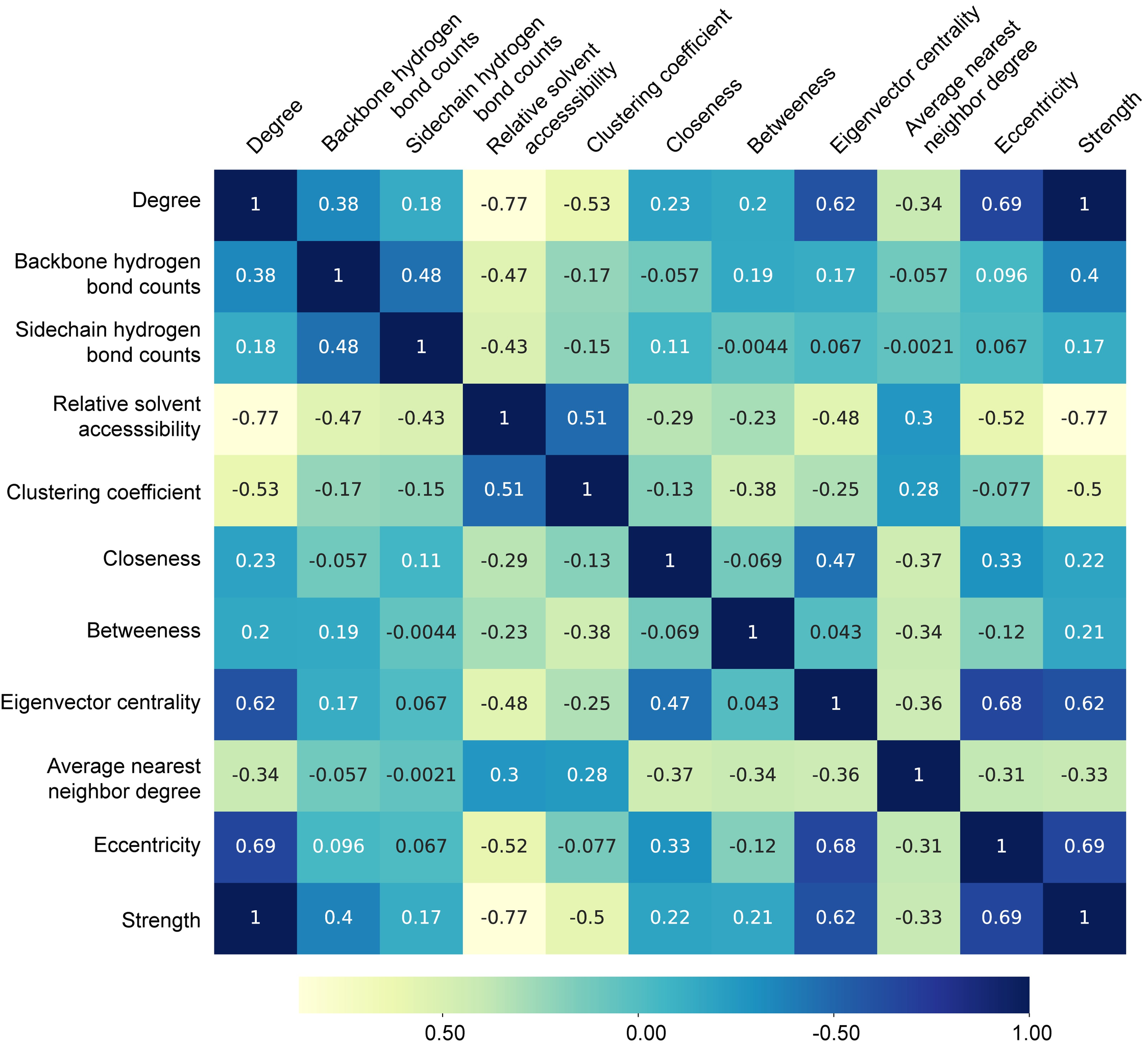

Supplement: S1 Fig — Values represent Pearson’s correlation. (TIF) [file pone.0299287.s001.tif]

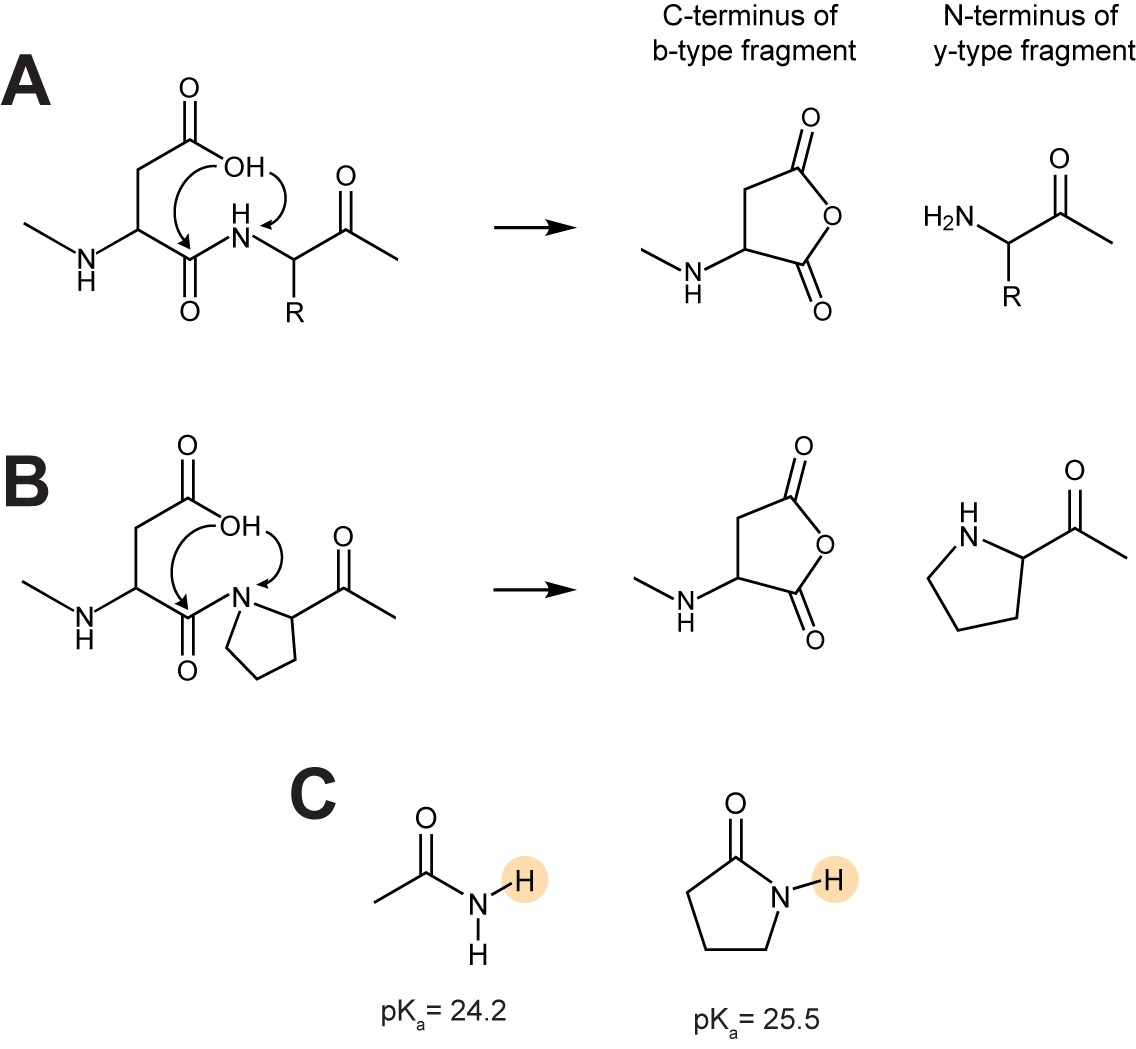

Supplement: S2 Fig — Comparison of the aspartic acid effect between D-residues with a C-terminal proline (P) residue (B) and those without (A). (A-B) The proposed aspartic acid effect mechanism [5]. (C) Theoretical pKa values of amine and imine in DMSO [46]. (TIF) [file pone.0299287.s002.tif]
