# Supplementary material for: Exploring the fragmentation efficiency of proteins analyzed by MALDI-TOF-TOF tandem mass spectrometry using computational and statistical analyses
Source: PLoS One. 2024 May 3;19(5):e0299287. doi: 10.1371/journal.pone.0299287 (PMC11068200; doi:10.1371/journal.pone.0299287)
Supplement: S1 Table — (DOCX) [file pone.0299287.s003.docx]

**S1 Table. Bacterial proteins analyzed by MALDI-TOF-TOF MS/MS.**

| **Bacterial strain** | **Protein** | **Sequence** | **Average theoretical MW (Da) of mature protein** | **Ref.** |
| --- | --- | --- | --- | --- |
| *E. coli* O113:H21  RM7788 | Immunity protein for Colicin E3 (Im3) (WP_000523346.1) | GLKLDLTWFDKSTEDFKGEEYSKDFGDDGSVMESLGVPFKDNVNNGCFDVIAEWVPLLQPYFNHQIDISDNEYFVSFDYRDGDW | 9771 | [1] |
| *E. coli* O113:H21 RM7807 | Bacteriocin immunity protein (WP_000421100.1) | MELKKSIGDYTETEFKKIIEDIINCEGDEKKQDDNLEYFINVTEHPSGSDLIYYPEGNNDGSPEGVIKEIKEWRAANGKSGFKQG | 9644 | [2] |
| E. coli O113:H21 RM7788 | Hypothetical protein (on plasmid), Imm Disulfide Intact (WP_000350688.1) | MDIEMNNTEEMFSKLGMRLGAEMAIPLKDGDNFAPTGCIVTKLDKENGLLICRPVQKEINGTWYTVDLSK | 7834 | [3] |
| *E. coli* O113:H21  RM7788 | Stress-induced protein, CsbD (YjbJ) | MNKDEAGGNWKQFKGKVKEQWGKLTDDDMTIIEGKRDQLVGKIQERYGYQKDQAEKEVVDWETRNEYRW | 8325 | [3] |
| *E. coli* O145:H28 RM13514 | Shiga-like toxin II subunit B precursor | ADCAKGKIEFSKYNEDDTFTVKVDGKEYWTSRWNLQPLLQSAQLTGMTVTIKSSTCESGSGFAEVQFNND | 7815 | [4] |
| *E. coli*  O145:H28  RM13514 | Acyl carrier protein (AHG14013.1) | STIEERVKKIIGEQLGVKQEEVTNNASFVEDLGADSLDTVELVMALEEEFDTEIPDEEAEKITTVQAAIDYINGHQA | 8508 | [4] |
| *E. coli*  O157:H7  RM6607W | Phosphocarrier protein (AHG15748.1) | MFQQEVTITAPNGLHTRPAAQFVKEAKGFTSEITVTSNGKSASAKSLFKLQTLGLTQGTVVTISAEGEDEQKAVEHLVKLMAELE | 9119 | [5] |
| *E. coli* phage  RM13514 | Phage tail fiber protein (AHG11868.1) | MNILKKLMQRLCGCGKHDDREHGELLTAQLRLGPADILESDENGIIPEQDRVITQVVILDADKKQIQCVVRPLQILRADGTWENIGGMK | 10000 | [4] |
| *E. coli* phage  RM13514 | Phage minor tail protein (AHG07267.1) | GLNANLKTYSVTLSVPREEATVLESFLEEHGGWKSFLWTPPYEWRQIKVTCAKWSSRVSMLRVEFSAEFEQ | 8281 | [4] |
| *E. coli*  RM13516 | HdeA (acid stress protein) (AHG11148.1) | ADAQKAADNKKPVNSWTCEDFLAVDESFQPTAVGFAEALNNKDKPEDAVLDVQGIATVTPAIVQACTQDKQANFKDKVKGEWDKIKKDM | 9738 | [4] |
| *E. coli*  RM13516 | HdeB (acid stress protein) (AHG11147.1) | ANESAKDMTCQEFIDLNPKAMTPVAWWMLHEETVYKGGDTVTLNETDLTQIPKVIEYCKKNPQKNLYTFKNQASNDLPN | 9063 | [4] |
| E. coli O157:H7 RM6607R | CsgA (curli monomer unit) | GVVPQYGGGGGNHGGGGNNSGPNSELNIYQYGGGNSALALQADARNSDLTITQHGGGNGADVGQGSDDSSIDLTQRGFGNSATLDQWNGKDSHMTVKQFGGGNGAAVDQTASNSTVNVTQVGFGNNATAHQY | 13143 | [6] |
| *Xyella fastidiosa* M23 | Chaperonin Cpn10 (ACB93030.1) | SIKPLHDRIVVKPIEADEVSPGGIVIPDSAKEKSTKGEVIAVGAGKPLDNGNVRTPCVKVGEKVIYGQYAGSTYKAEGVEYKVLREDDILAIIG | 9960 | [7] |
| *Xyella fastidiosa* M23 | Histone family protein DNA-binding protein (ACB91917.1) | MNKTELIDGVAAAANLSKVEAGRAIDAVVNEITEALKEGDSVTLVGFGTFQVRQRAERPGRNPKTGEPIMIAASNNPSFKPGKALKDAVKSSAG | 9780 | [7] |
| *Xyella fastidiosa* M23 | Cold-shock DNA-binding domain protein (ACB93496.1) | SDTSDDRQIGTVKWFNDNKGFGFITSNNGPDLFVHYRAIQGNGFKSLQEGQKVSFVAVQGQKGMQADQVQVL | 7950 | [7] |
| *Xyella fastidiosa* M23 | Hypothetical protein XfasM23_0565 (ACB92009.1) | QAPKASSTATEMQQPASPSTDMPTNSEGQGMGEHTGEHKVRKSKKHRSHSAKPMSTGSATAPTKEAEENTSATR | 7775 | [7] |
| *Xyella fastidiosa* M23 | Cold-shock DNA-binding domain protein (ACB92876.1) | MQSGTVKWFSDQKGFGFILPDDGTPEVFVHYSGINSKGFRSLHEGQRVTYDVTQGPKGPQASNITPI | 7357 | [7] |
| *Xyella fastidiosa* strain M23 | Ribosomal protein L33 (ACB91929.1) | AGKRDKIRLISSADTGHFYTTDKNKKNTPGKLEFKKYDPRVRRHVIYKEGKIK | 6234 | [7] |
| *Xyella fastidiosa* strain M23 | Outer membrane protein (ACB91756.1) | CGSKTPETPPTQDPNPASNPAANEAQAAADQAAANPPADATPAAADTTAAAANTAANAAATATPPADAAKQSGAAPATPATKAKNPAEQKK | 8555 | [7] |
| *Xyella fastidiosa* strain Stags Leap | Hypothetical protein (WP_004088076.1) | CGSNTPEKPPTQDPNPPSNPAANEAQAAADQAAANPPADATPAAADTAAAAANTAADAAATTTPPADAAKQSGAAPANKAKNPAEQKK | 8339 | [7] |
| *E.coli* O113:H21 RM78077 | Cold-shock protein C (CspC) | AKIKGQVKWFNESKGFGFITPADGSKDVFVHFSAIQGNGFKTLAEGQNVEFEIQDGQKGPAAVNVTAI | 7271 | [2] |
| *E.coli* O113:H21 RM78077 | Cold-shock protein E (CspE) | SKIKGNVKWFNESKGFGFITPEDGSKDVFVHFSAIQTNGFKTLAEGQRVEFEITNGAKGPSAANVIAL | 7332 | [2] |
| *E.coli* O145:H28 RM13514 | DNA-binding protein HU-α | MNKTQLIDVIAEKAELSKTQAKAALESTLAAITESLKEGDAVQLVGFGTFKVNHRAERTGRNPQTGKEIKIAAANVPAFVSGKALKDAVK | 9535 | [5] |
| *E.coli* O157:H7 RM13516 | DNA-binding protein HU-β | MNKSQLIDKIAAGADISKAAAGRALDAIIASVTESLKEGDDVALVGFGTFAVKERAARTGRNPQTGKEITIAAAKVPSFRAGKALKDAVN | 9225 | [5] |
| *E.coli* O157:H7 RM6607 | Osmotically inducible protein OsmY | ENNAQTTNESAGQKVDSSMNKVGNFMDDSAITAKVKAALVDHDNIKSTDISVKTDQKVVTLSGFVESQAQAEEAVKVAKGVEGVTSVSDKLHVRDAKEGSVKGYAGDTATTSEIKAKLLADDIVPSRHVKVETTDGVVQLSGIVDSQAQSDRAESIAKAVDGVKSVKNDLKTK | 18173 | [8] |
| *E.coli* O157:H7 RM6607 | Osmotically inducible protein OsmY (with C-terminal truncation) | ENNAQTTNESAGQKVDSSMNKVGNFMDDSAITAKVKAALVDHDNIKSTDISVKTDQKVVTLSGFVESQAQAEEAVKVAKGVEGVTSVSDKLHVRDAKEGSVKGYAGDTA | 11399 | [8] |
| *E.coli* O157:H7 strain EDL933 | YahO orf, hypothetical protein | AELMTKAEFEKVESQYEKIGDISTSNEMSTADAKEDLIKKADEKGADVLVLTSGQTDNKIHGTADIYKKK | 7708 | [9] |
| *Campy. upsaliensis* RM3195 | Thioredoxin | GKYIELTSENFATAKEGVALVDFWAPWCGPCKMLSPVIDELASDFEGKAKICKVNTDEQGDLAAEYGVRSIPTLIFFKNGEVVGQLVGAQSKQTIADKINSLL | 11137 | [10] |
| *Campy. upsaliensis* RM3195 | Ribosomal protein L7/L12 | AISKEDVLEYISNLSVLELSELVKEFEEKFGVSAAPVVVAGGAAAGGGAAAAEEKTEFDIVLTDSGAKKIEVIKIVRALTGLGLKEAKDAVEQTPSTLKEGVAKADAEEAKKQLEEAGAKVELK | 12855 | [10] |
| *Campy. upsaliensis* RM3195 | 10 kDa chaperonin | MNFQPLGKRVLVKRVEETKTTASGIIIPDNAKEKPLIGEVVAVSKEVSDIASGDKIVFAKYGGTEVKLNDGEYLVLNLDDVLGILK | 9300 | [10] |
| *Campy. upsaliensis* RM3195 | Protein of unknown function (DUF465) family | MLHEFRDLMSELKGKDAHFDKLFERHNELDDKIKDAEEGRAFLSDVEISTLKKEKLHVKDELAQYLANYKK | 8450 | [10] |
| *Campy. coli* RM2228 | Protein of unknown function (DUF465) family | MLHEYRELMSELKGKDAHFDKLFERHNELDDQIKDAEEGRNLLSDIEISNLKKEKLHIKDQLNQYLANYKK | 8572 | [10] |
| *Campy. coli* RM2228 | Ribosomal protein L7/L12 | AISKEDVLEYISNLSVLELSELVKEFEEKFGVSAAPVMIAGGAAAGGAAAAAEEKTEFDIVLTDGGAKKIEVIKIVRALTGLGLKEAKDAVEQTPSTLKEGVAKAEAEEAKKQLEEAGAKVELK | 12854 | [5] |
| *Campy. jejuni* RM1221 | Ribosomal protein L7/L12 | AISKEDVLEYISNLSVLELSELVKEFEEKFGVSAAPVMIAGGAAAGGAAAAAEEKTEFDIVLTDGGAKKIEVIKIVRALTGLGLKEAKDAVEQTPSTLKEGVAKAEAEEAKKQLEEAGAKVELK | 12900 | [5] |
| *Salmonella enterica* Infantis 119944 | YahO Hypothetical protein L287_16136 | AEIMKKTDFDKVASEYTKIGTISTTGEMSPLDAREDLIKKADEKGADVVVLTSGQTENKIHGTADIYKKK | 7660 | [5] |
| *E. coli* O178 RM13956 on plasmid | Immmunity protein E8 | MELKNSISDYTETEFKKIIEDIINCEGDEKKQDDNLEHFISVTEHPSASDLIYYPEGNNDGSPEAVIKEIKEWRAANGKSGFKQG | 9635 | [5] |

**References**

1. Fagerquist CK, Rojas E. Identification of Antibacterial Immunity Proteins in Escherichia coli using MALDI-TOF-TOF-MS/MS and Top-Down Proteomic Analysis. J Vis Exp. 2021; e62577. doi:10.3791/62577

2. Fagerquist CK, Dodd CE. Top-down proteomic identification of plasmid and host proteins produced by pathogenic Escherichia coli using MALDI-TOF-TOF tandem mass spectrometry. PLOS ONE. 2021;16: e0260650. doi:10.1371/journal.pone.0260650

3. Fagerquist CK, Lee BG, Zaragoza WJ, Yambao JC, Quiñones B. Software for top-down proteomic identification of a plasmid-borne factor (and other proteins) from genomically sequenced pathogenic bacteria using MALDI-TOF-TOF-MS/MS and post-source decay. Int J Mass Spectrom. 2019;438: 1–12. doi:10.1016/j.ijms.2018.12.006

4. Fagerquist CK, Shi Y, Dodd CE. Toxin and phage production from pathogenic E. coli by antibiotic induction analyzed by chemical reduction, MALDI-TOF-TOF mass spectrometry and top-down proteomic analysis. Rapid Commun Mass Spectrom. 2023;37: e9505. doi:10.1002/rcm.9505

5. Park J, Fagerquist CK. MS/MS of specific protein biomarkers of pathogenic bacteria. doi:10.5281/zenodo.8190267

6. Fagerquist CK, Shi Y. Biofilm of pathogenic bacteria analyzed by MALDI-TOF imaging mass spectrometry (IMS) and top-down proteomic identification. Proceedings of 71st ASMS Conference on Mass Spectrometry and Allied Topics;

7. Fagerquist CK, Wallis CM, Chen J. Top-down proteomic identification of protein biomarkers of Xylella fastidiosa subsp. fastidiosa using MALDI-TOF-TOF-MS and MS/MS. Int J Mass Spectrom. 2023;489: 117051. doi:10.1016/j.ijms.2023.117051

8. Fagerquist CK, Shi Y, Park J. Unusual modifications of protein biomarkers expressed by plasmid, prophage, and bacterial host of pathogenic Escherichia coli identified using top-down proteomic analysis. Rapid Commun Mass Spectrom. 2024;38: e9667. doi:10.1002/rcm.9667

9. Fagerquist CK, Garbus BR, Miller WG, Williams KE, Yee E, Bates AH, et al. Rapid Identification of Protein Biomarkers of Escherichia coli O157:H7 by Matrix-Assisted Laser Desorption Ionization-Time-of-Flight−Time-of-Flight Mass Spectrometry and Top-Down Proteomics. Anal Chem. 2010;82: 2717–2725. doi:10.1021/ac902455d

10. Fagerquist Clifton K., Garbus Brandon R., Williams Katherine E., Bates Anna H., Boyle Síobhán, Harden Leslie A. Web-Based Software for Rapid Top-Down Proteomic Identification of Protein Biomarkers, with Implications for Bacterial Identification. Appl Environ Microbiol. 2009;75: 4341–4353. doi:10.1128/AEM.00079-09
